# Coping Strategies, ACEs, and Mental Health Among Adolescents with Child Welfare Involvement

**DOI:** 10.1007/s40653-026-00874-7

**Published:** 2026-04-13

**Authors:** Yunqi He, Lindsey M. Weiler, Eunyoung Park, Haoran Zhou, Ana Mireya Díaz, Heather N. Taussig

**Affiliations:** 1 Department of Family Social Science, University of Minnesota, Minneapolis, United States; 2 Graduate School of Social Work, University of Denver, Denver, United States; 3 Kempe Center, University of Colorado, Aurora, United States

**Keywords:** Coping strategies, Child welfare, Mental health, ACEs, Adolescence

## Abstract

Adolescents involved in the child welfare system face disproportionate mental health risks, yet our understanding of how coping strategies function within this population remains limited. This cross-sectional study examined how various coping strategies relate to mental health functioning among 245 adolescents with child welfare involvement and whether these relationships were moderated by adverse childhood experiences (ACEs). Using a modified version of the Children’s Coping Strategies Checklist and the Trauma Symptom Checklist for Children, hierarchical regression analyses revealed both direct associations and significant moderation effects. Direct effects showed that positive cognitive restructuring was associated with lower anxiety, depression, and posttraumatic stress symptoms, and problem-focused coping was related to fewer anger symptoms. Additionally, avoidance and distraction coping were associated with higher symptoms across multiple mental health concerns. However, simple slopes analyses revealed that ACEs altered these associations. Problem-focused coping showed negative associations with anger, dissociation, and sexual concerns at high ACE levels but not at mean or low ACE levels, and avoidance coping was positively associated with anxiety and depression at high and mean ACEs levels but not at low ACE level. Two strategies typically associated with fewer mental health problems showed a different pattern at high ACE levels (while showing no association at mean or low ACE levels): support-seeking was associated with increased anger symptoms and cognitive restructuring with increased sexual concerns. These findings highlight the context-dependent nature of coping and mental health associations and underscore the need for trauma-informed interventions that consider both adversity exposure and the development of coping skills tailored to adversity levels and available resources.

## Introduction

Coping strategies, the behavioral and cognitive efforts individuals employ to manage stressful situations, have been identified as critical determinants of mental health outcomes for youth (Compas et al., 2017). Research consistently demonstrates that adolescents who employ certain coping approaches, such as problem-solving and cognitive reframing, tend to experience better mental health outcomes than those who rely on avoidant strategies (Cicognani, 2011; Compas et al., 2001). Variation in coping strategies and their association with mental health problems has led researchers to examine factors that contribute to this relationship, including individual characteristics, environmental contexts, and exposure to stress (Perzow et al., 2021). However, most adolescent coping research has focused on general populations. While some studies have examined coping patterns among youth involved in the child welfare, few have investigated how the relationships between specific coping strategies and mental health problems may differ in this population or whether these associations vary by adversity levels, leaving important questions about how these protective processes function in higher risk populations.

For adolescents involved in the child welfare system, understanding their coping approaches is particularly crucial given their elevated risk for mental health problems. Youth with child welfare involvement experience significantly higher rates of anxiety, depression, and posttraumatic stress symptoms compared to community samples (Bronsard et al., 2016; Dubois-Comtois et al., 2021). These mental health challenges occur within the context of complex adverse experiences, including maltreatment, family separation, placement instability, and other system-related stressors (Bederian-Gardner et al., 2018; Greeson et al., 2011). These unique challenges may fundamentally alter how coping strategies relate to mental health, suggesting that findings from community samples may not generalize to this population (Jackson et al., 2017).

Importantly, the level of adversity experienced varies considerably among child welfare-involved youth. Some face discrete maltreatment incidents, while others endure chronic and complex trauma across multiple domains. This raises important questions about whether the relationship between coping approaches and mental health problems varies by adversity childhood experiences (ACEs), a possibility that has received limited empirical attention. This study addresses these research gaps by examining how various coping strategies relate to mental health problems among adolescents with child welfare involvement in a large Western U.S. city and explores whether these relationships are moderated by the level of ACEs. While prior research has examined coping and mental health in child welfare populations (e.g., Jackson et al., 2017; Kothari et al., 2020), and one study tested whether coping moderates the maltreatment–mental health relationship (Huffhines et al., 2020), research specifically examining whether adversity level moderates the relationship between coping strategies and mental health outcomes remains limited. Additionally, the current study uses an empirically derived ACEs index developed specifically for adolescents involved in the child welfare system (Weiler et al., 2025), which captures a broader range of adversities than traditional ACE measures, including maltreatment, witnessing violence, child welfare system involvement, instability, and peer violence. This is among the first studies to explicitly test whether the effectiveness of different coping strategies varies by level of cumulative adversity among adolescents with child welfare involvement.

## Literature Review

### The Response to Stress Framework for Understanding Coping in Adolescence

The Responses to Stress (RTS; Connor-Smith et al., 2000) framework categorizes coping strategies into three domains. *Primary Control Engagement Coping* (PCEC) includes active strategies aimed at directly addressing the stressor or one’s emotional response to it, such as problem-solving and seeking social support. *Secondary Control Engagement Coping* (SCEC) involves efforts to adapt oneself to stressful conditions through cognitive and emotional regulation strategies, with positive cognitive restructuring being a central strategy. Rather than attempting to change the stressor directly, the individual focuses on adapting their thoughts, feelings, or behaviors to better manage the stress. *Disengagement Coping* (DC) refers to strategies that involve mentally or behaviorally withdrawing from stress, such as avoiding the problem (i.e., avoidance coping) or distracting oneself from distressing thoughts and feelings (i.e., distraction coping). These approaches create distance between the individual and the stressor rather than actively addressing or adapting to the situation. This framework shares conceptual foundations with Compas et al.‘s (2017) Control-Based Model of Coping, which similarly distinguishes between primary control, secondary control, and disengagement responses. The RTS framework was selected for this study because it directly informed the development of the coping measure used, ensuring alignment between theoretical and measurement approaches.

It is important to note that coping and emotion regulation are closely related but distinct constructs. Following Compas et al. (2017), emotion regulation can be understood as a subset of coping efforts specifically aimed at modulating emotional responses. For its purposes, this study focuses on coping as the broader construct encompassing cognitive and behavioral efforts to manage stressful situations, which includes but is not limited to emotion regulation strategies.

Research with general adolescent populations has shown that engagement strategies (both PCEC and SCEC) are typically associated with better psychological adjustment, including lower levels of internalizing and externalizing symptoms (Compas et al., 2017; Skinner & Zimmer-Gembeck, 2016). Conversely, DC strategies have been linked to poorer psychological outcomes, including increased anxiety, depression, and behavioral problems in general adolescent samples (Seiffge-Krenke, 2011; Wadsworth & Compas, 2002). However, this pattern may manifest differently with youth who have experiences a high number of ACEs and warrants empirical investigation.

### Developmental Considerations for Adolescents with Child Welfare Involvement

Adolescence represents a critical developmental period marked by significant neurobiological changes, including ongoing prefrontal cortex maturation and heightened limbic system reactivity. These changes can manifest behaviorally as intensified emotional experiences and/or still-developing self-regulation capacities (Arain et al., 2013; Maciejewski et al., 2015). For adolescents exposed to significant adversity, these normative developmental processes become substantially more complex. Because of the adolescent brain’s heightened stress sensitivity, chronic adversity can disrupt normal neural development, particularly in brain regions responsible for executive functioning and emotional regulation (Teicher et al., 2016). This neurobiological vulnerability is particularly pronounced for youth receiving child welfare services, who need to simultaneously navigate typical adolescent developmental tasks while managing previous trauma exposure, placement instability, and frequent disruptions to supportive relationships (Goemans et al., 2016). While placement instability is a significant concern for youth in out-of-home care, many child welfare-involved youth remain in their homes while receiving services. These youth may face ongoing family stressors, system-related demands, and other adversities that similarly constrain coping development. It is therefore important to consider the full range of adversities experienced by child welfare-involved youth rather than focusing exclusively on out-of-home placement experiences. Across these varied contexts, environmental constraints limit opportunities to develop and practice adaptive coping strategies in multiple ways. First, the unstable and under-resourced contexts characteristic of child welfare involvement often lacks the foundational elements—such as consistent supportive relationships, predictable routines, and accessible resources—that enable youth to learn and implement evidence-based coping approaches (Bederian-Gardner et al., 2018; Grey et al., 2015). Second, even when youth are aware of potentially adaptive strategies, their environments may render these approaches practically unfeasible; for example, seeking social support becomes difficult when relationships are frequently disrupted, and maintaining stability-dependent routines is challenging when living situations are unpredictable (Seita et al., 2016). Together, these barriers suggest that traditional assumptions about adaptive coping derived from community-based samples may not apply to youth whose developmental trajectories have been fundamentally shaped by adversity and system involvement (Jackson et al., 2017).

#### Primary Control Engagement Coping in Child Welfare Contexts

For youth involved with the child welfare system who experience caregiving instability and/or limited social resources, Primary Control Engagement Coping (PCEC) strategies like problem-solving and support-seeking may be less accessible (Konijn et al., 2019; Seita et al., 2016). Research on coping patterns in child welfare-involved populations reveals important nuances in how these strategies are employed and their effectiveness. Among youth in foster care, research demonstrates notably high endorsement of direct-action coping strategies, which align closely with PCEC approaches. Using the Behavioral Inventory of Strategic Control (BISC; Little et al., 2001; Lopez & Little, 1996), which categorizes coping along four dimensions: direct action (moving toward the stressor), indirect action (avoiding the stressor), prosocial action (seeking help from others), and asocial action (handling problems alone), Jackson et al. (2017) found that over 50% of youth in foster care showed a preference for direct coping approaches based on their highest reported use scores compared to other coping styles across multiple time points. However, the effectiveness of PCEC strategies in child welfare contexts appears to be nuanced and context-dependent. While direct problem-solving has been associated with lower caregiver-reported internalizing and externalizing symptoms among youth in foster care (Huffhines et al., 2020), the controllability of stressors matters significantly. Clarke’s (2006) meta-analysis revealed that PCEC strategies were beneficial for controllable stressors such as arguments with peers or conflicts with siblings, but less effective for uncontrollable stressors such as parental discord or abuse. The distinction between controllable and uncontrollable stressors is relevant for child welfare-involved youth who may face myriad situations beyond their control.

Social dimensions of PCEC present additional considerations, as dealing with problems alone has been linked to higher self-reported internalizing symptoms and more psychiatric hospitalizations in foster care samples (Huffhines et al., 2020). In contrast, support-seeking strategies can have protective effects on anxiety and depression symptoms among youth who age out of foster care, though having low social support resources can have the opposite effect, highlighting the moderating role of available resources (Grey et al., 2015, p. 201). However, the child welfare experience may compromise youth’s orientation toward support-seeking within their social networks. Seita et al. (2016) found that longer lengths of stay in foster care, higher numbers of placements, and lack of permanency arrangements were all associated with decreased likelihood of asking for and accepting assistance from social networks, with placement instability being the strongest predictor of reduced social network orientation.

The challenges with support-seeking, combined with the mixed effectiveness of problem-solving approaches, reflect both developmental and contextual factors specific to child welfare involvement. For some youth, inconsistent caregiving situations may limit opportunities to observe and practice effective problem-solving approaches through modeling and guided practice (Kemmis-Riggs et al., 2018), potentially affecting their ability to develop and implement such strategies when facing new challenges. This developmental limitation can be particularly problematic for PCEC strategies that rely on learned problem-solving skills and confidence in one’s ability to effect change.

#### Secondary Control Engagement Coping in Child Welfare Contexts

Research specifically examining Secondary Control Engagement Coping (SCEC) strategies among youth with child welfare involvement is limited. However, available evidence suggests that the associations between SCEC strategies and mental health outcomes may differ for youth with maltreatment histories, as the cognitive and emotional processing capacities necessary for effective SCEC strategies may be particularly compromised in this population (Teicher et al., 2016; Tottenham & Galván, 2016). Research has indicated that maltreatment affects fundamental cognitive processes including deploying attention, integrating information, and making judgments and decisions (Dvir et al., 2014). Childhood adversity may alter cognitive appraisals related to threat, making it difficult for youth to generate alternative, adaptive interpretations of stressful situations (Teicher et al., 2016). Specific attention difficulties have been documented, with some children who experienced physical abuse showing problems disengaging attention from angry facial cues and demonstrating rumination and attention bias toward negative stimuli (Dvir et al., 2014). These attention biases could interfere with cognitive reframing efforts, potentially weakening the protective associations between positive cognitive restructuring and mental health outcomes that are typically observed in general adolescent samples. Complementing these cognitive processing difficulties, maltreated children also demonstrate significant deficits in emotional understanding that could impair SCEC effectiveness. Girls who experienced sexual abuse, for example, were less able to understand emotions and expected to encounter more interpersonal conflict in response to expressing negative emotional states than non-maltreated girls (Caouette et al., 2023; Shipman et al., 2000). Similarly, children who experienced neglect showed decreased understanding of negative emotions such as anger and sadness as compared to their non-maltreated peers (Shipman et al., 2005). Together, these cognitive and emotional processing disruptions suggest that even when youth with maltreatment histories employ SCEC strategies, the expected protective associations with mental health outcomes may be attenuated or, in some cases, reversed.

#### Disengagement Coping in Child Welfare Contexts

DC strategies present an ever more complex picture among youth with child welfare involvement, with research revealing both adaptive and maladaptive functions depending on context and individual factors. While traditional perspectives have viewed avoidance and disengagement as primarily maladaptive, emerging evidence suggests these strategies may serve protective functions in certain child welfare contexts (Clarke, 2006; Elzy et al., 2013), particularly when youth face uncontrollable stressors. For instance, Huffhines et al. (2020) found that indirect coping, which defined as behavioral strategies that move away from rather than toward stressors (similar to DC strategies), was associated with fewer psychiatric hospitalizations, particularly among youth with high maltreatment chronicity. While indirect coping represents one dimension of the broader DC construct, this finding aligns with other research demonstrating protective effects of avoidant strategies. Specifically, youth with extensive maltreatment histories who employed avoidant strategies showed fewer psychiatric hospitalizations compared to those who did not use these approaches when facing severe, uncontrollable stressors. Similarly, Chaffin et al. (1997) and Elzy et al. (2013) found that avoidance coping was associated with fewer internalizing and externalizing symptoms among sexually abused youth, suggesting that withdrawal from overwhelming stressors may be adaptive in certain contexts.

This pattern may reflect the learned coping responses that youth developed during their traumatic experiences, prior to child welfare involvement. When youth previously lived in abusive environments, direct engagement was often neither possible nor safe- a child could not directly solve parental substance abuse, stop physical abuse, or change dangerous living conditions. In these contexts, disengagement strategies such as avoidance, distraction, or emotional withdrawal may have been adaptive and even protective responses that helped youth survive overwhelming and uncontrollable circumstances (Compas et al., 2001). These learned coping patterns may persist even after youth enter safer environments. However, the effectiveness of these strategies appears to depend critically on the specific mental health presentation and contextual factors. This complexity helps explain why youth in child welfare settings show high reliance on DC strategies despite mixed outcomes in the literature. Given the elevated rates of anxiety and depression in this population, direct engagement with stressors may feel overwhelming, making DC strategies appear more manageable in the short term, even when they may not promote optimal long-term adjustment across all domains of functioning.

### Adversity and Coping in Child Welfare Contexts

Given the unique developmental and environmental challenges faced by this population, it is possible that the level of adversity experienced may also influence the effectiveness of different coping approaches. To better understand these coping patterns, adverse experiences are often measured using indices of Adverse Childhood Experiences (ACEs), which assess the cumulative exposure to different types of adversity. For adolescents involved in the child welfare system, expanded ACEs indices that capture a wider range of adversities, such as parental rights termination, caregiver changes, school transitions, and unmet basic needs, may be more helpful (Raviv et al., 2010; Shipman et al., 2005; Weiler et al., 2025). These expanded measures are crucial because child welfare-involved youth typically experience adversities beyond the traditional ACEs categories, including system-specific stressors like placement instability, separation from siblings, and navigating multiple caregiving relationships.

The chronicity and cumulative nature of adversity are important for understanding both coping strategy selection and mental health problems, as youth who experience more frequent maltreatment have shown different coping patterns and adjustment trajectories than those with less chronic exposure (Huffhines et al., 2020). The impact of adversity on coping and mental health problems can be understood through several interconnected processes. First, repeated exposure to stress can dysregulate neurobiological stress response systems, affecting emotional processing and regulation (Dvir et al., 2014; McLaughlin & Lambert, 2017). This neurobiological impact may alter how effectively youth can employ various coping strategies, particularly those requiring emotional awareness and regulation. Additionally, youth who experience chronic adversity may be more likely to exhibit inactive or low-engagement coping patterns (Perzow et al., 2021). Research with youth in foster care supports this pattern, showing that those with high maltreatment chronicity may actually benefit from indirect/avoidant coping strategies by experiencing fewer psychiatric hospitalizations and reduced trauma symptoms when facing severe, uncontrollable stressors (Elzy et al., 2013; Huffhines et al., 2020). These interconnected effects suggest that as adversity accumulates, the relationship between coping strategies and mental health outcomes may shift, with certain strategies becoming less effective or counterproductive in high-adversity contexts, while others that are typically considered maladaptive may serve protective functions. However, this nuanced understanding of context-dependent coping effectiveness requires further empirical investigation.

## Current Study

Previous research has shown that coping strategies are associated with mental health outcomes in general adolescent populations, with engagement strategies linked to better psychological functioning and disengagement strategies associated with greater distress (Compas et al., 2017). However, less is known about how these coping strategies operate in adolescents with child welfare involvement, and few studies have considered whether the associations between these strategies and mental health outcomes vary by the level of adversity experienced.

The study hypotheses include:

1. Primary Control Engagement Coping (i.e., problem-focused coping, support-seeking) will be negatively associated with mental health problems.
2. Secondary Control Engagement Coping (i.e., positive cognitive restructuring) will be negatively associated with mental health problems.
3. Disengagement Coping (i.e., avoidance, distraction) will be positively associated with mental health problems.
4. The level of adversity experienced will moderate the relationships between coping strategies and mental health problems. Given the limited prior research specifically examining adversity as a moderator of coping-outcome relationships in this population (Huffhines et al., 2020; Stapley et al., 2023), and the conflicting findings in the existing literature regarding coping effectiveness in high-adversity contexts, specific patterns of these moderation effects will be examined exploratorily to identify patterns that can inform future confirmatory research.

## Method

### Recruitment

This cross-sectional study used baseline data from the *Fostering Healthy Futures for Teens* (FHF-T) study (Taussig et al., 2020), a randomized controlled trial of a preventive intervention for adolescents with child welfare involvement in a large Western U.S. city. FHF-T is an adaptation of the evidence-based Fostering Healthy Futures program (Taussig & Culhane, 2010; Taussig et al., 2012), providing 30 weeks of one-on-one mentoring by graduate students in social work and related fields, with individualized skills training across five domains (Taussig et al., 2015). All data used in the current study were collected pre-randomization across four cohorts over four consecutive summers from 2015 to 2018, and therefore the intervention does not affect the current findings. Youth were eligible for the study if they were entering 8th or 9th grade and had an open child welfare case due to maltreatment in one of four participating metropolitan counties included in the study. All youth who met these criteria were recruited with the exception of youth who lived more than a 35-minute drive from the program site, as well as youth with significant developmental delays, those who had been adjudicated for a sexual or violent offense (i.e., as perpetrators), or those who were expecting/parenting a child (all based on caseworker report). Initially, 327 youth were assessed for eligibility; 21 were excluded as they did not meet the inclusion criteria and 61 did not consent. As such, a total of 245 youth (80.1% recruitment rate) was eligible and agreed to participate.

### Procedure

Informed assent and caregiver consent were obtained prior to data collection. Participation in the study was voluntary and could not be court-ordered. Letters introducing the study were sent to families. Youth were interviewed individually and paid $50.00/interview. The university’s Institutional Review Board approved the study protocol.

### Participants

Less than half (38.8%) of participants were male and 61.2% were female. Participants ranged in age from approximately 13 to 16 years (*M* = 13.99, *SD* = 0.69). Participants’ ethnic and racial identity (non-exclusive categories) were: White (51%), Hispanic (48.6%), Black/African American (24.9%), Native American (25.7%), Asian (2%), and Pacific Islander (0.8%). Over half (55.1%) were living at home and 44.9% were in out-of-home care (e.g., kinship, foster, group home) at the time of data collection.

### Measures

#### Youth’s Coping Strategies

A modified version of the 52-item Children’s Coping Strategies Checklist (CCSC; Program for Prevention Research, 1999) was used to measure coping strategies. Total items were reduced to prevent overburden and fatigue among participants (Chesmore et al., 2017). The modified CCSC-R scale comprised five subscales: Problem-focused coping (6 items; α = 0.80; *M* = 2.53, *SD* = 0.59; e.g., “Think about what you can do before you do anything”), Positive cognitive restructuring (8 items; α = 0.81; *M* = 2.55, *SD* = 0.57; e.g., “Tell yourself that things will get better”), Support-seeking coping (4 items; α = 0.77; *M* = 2.36, *SD* = 0.74; e.g., “Talk to someone who can help you figure out what to do”), Distraction coping (7 items; α = 0.67; *M* = 2.59, *SD* = 0.56; e.g., “Listen to music, watch TV, or read a book”), and Avoidance coping (6 items; α = 0.69; *M* = 2.39, *SD* = 0.59; e.g., “Try to stay away from the problem”). Items were rated on a 4-point scale (1 = *Never* to 4 = *Most of the time*). The interviewer introduced the measure by saying: “Sometimes kids have problems or feel upset about things. When this happens, they may do different things to solve the problem or to make themselves feel better. For each item below, choose the answer that best describes how often you usually do this to solve your problems or make yourself feel better.” Participants’ responses were recorded on a scale ranging from 1 (*Never*) to 4 (*Most of the time*). Higher mean scores indicate greater frequency of the strategy. The CCSC has been used with child welfare-involved and maltreated youth populations (e.g., Chesmore et al., 2017) and the modified version demonstrated adequate internal consistency in the current sample across subscales (α = 0.67–0.81).

#### Mental Health Problems

The Trauma Symptom Checklist for Children (TSCC; Briere, 1996) was used to evaluate participants’ mental health. It encompasses six clinical subscales: Anxiety, Depression, Anger, Posttraumatic Stress, Dissociation, and Sexual Concerns. This 54-item self-report measure was administered to participants via structured interviews. Participants were provided with these instructions: “The following sentences describe things that kids sometimes think, feel, or do. Please tell me how often each thing happens to you.” Responses to each item were recorded on a scale ranging from 0 (*Never*) to 3 (*Almost all of the time*). Each subscale yielded a standardized *T*-score, with higher scores suggesting greater symptoms. The TSCC has demonstrated strong psychometric properties across diverse trauma-exposed populations, including psychiatrically hospitalized adolescents (Sadowski & Friedrich, 2000), juvenile justice-involved youth (Kretschmar et al., 2018), and children evaluated at child advocacy centers (Wherry & Dunlop, 2018). Across studies, Cronbach’s alpha coefficients for the clinical scales typically range from 0.70 to 0.90 (Briere, 1996; Sadowski & Friedrich, 2000). In the current sample, internal consistency was as follows: Anxiety (α = 0.83), Depression (α = 0.88), Anger (α = 0.88), Posttraumatic Stress (α = 0.87), Dissociation (α = 0.85), and Sexual Concerns (α = 0.77).

#### Demographic Characteristics

Youth’s sex (0 = *Female*, 1 = *Male*), age, and ethnicity/race were collected via self-, caregiver-, and caseworker-reports as well as child welfare records. Group differences by Asian and Pacific Islander identities were not examined due to low representation in the sample. Number of referrals to child protective services and number of previous dependency and neglect (D&N) cases were extracted from administrative records.

#### Adverse Childhood Experiences

An ACEs index (Weiler et al., 2025) was used to assess youth adversity. This empirically derived 14-item index was developed using the same sample as the current study to capture a comprehensive range of adverse experiences relevant for adolescents involved in the child welfare system. The index includes items related to maltreatment, witnessing violence, child welfare system involvement, instability (i.e., number of caregiver transitions, number of school changes), and peer and relationship-based violence.

Data for the ACEs index were collected through multiple sources, including Child Protection Services intake reports, youth self-reports, and administrative data. Maltreatment history was coded from Child Protection Services’ intake reports by trained research assistants using a modified version of the Maltreatment Classification System (Barnett et al., 1993; inter-rater agreement κ = 0.60–1.0). Termination of parental rights was assessed via caseworker reports at study recruitment. The remaining items were assessed through youth and caregiver interviews using established measures: witnessing domestic violence (Revised Conflict Tactics Scale; Straus et al., 1996), witnessing community violence (Things I Have Seen and Heard scale; Richters & Martinez, 1993; α = 0.77), peer emotional bullying, peer physical bullying, and physical violence due to discrimination (Juvenile Victimization Questionnaire; Hamby et al., 2004), cyberbullying (project-designed measure), and emotional/relational and sexual/physical dating violence (Conflict in Adolescent Dating Relationships Inventory; Wolfe et al., 2001). Number of caregiver transitions was constructed from youth interview data, and number of school changes was youth-reported. Consistent with previous cumulative risk studies (Appleyard et al., 2005; Finkelhor et al., 2013; Raviv et al., 2010), each item was dichotomized (0 = not experienced, 1 = experienced), with continuous variables dichotomized at the upper quartile of the sample distribution. A mean score was calculated across the 14 items to account for missing data and multiplied by 14 to create a total ACEs score (Weiler et al., 2025). For prevalence rates of each individual adversity, see Weiler et al. (2025, Table 1). In the current sample, adolescents reported experiencing an average of 3.2 ACEs (*SD* = 2.18, range 0–10).

**Table 1:** Correlations Between Demographic Variables, Coping Strategies, and Mental Health Outcomes

Variable	Age	Sex (male)	Hispanic or Latinx	African American	Caucasian	American Indian	Number of Referrals	Number of Previous D&Ns
Coping Strategy								
Problem-focused Coping	− 0.06	0.02	0.00	0.14*	0.00	0.00	0.04	− 0.04
Support-Seeking	0.03	− 0.03	0.05	0.03	0.02	− 0.02	0.10	0.00
PC Restructuring	− 0.02	0.03	0.04	0.10	0.05	0.04	0.05	− 0.12
Distraction Coping	− 0.03	− 0.01	0.02	0.15*	− 0.03	0.07	0.03	0.00
Avoidance Coping	− 0.11	− 0.08	0.07	0.05	− 0.07	0.02	0.04	− 0.13
Mental Health Problem								
Anxiety	0.01	− 0.04	0.00	0.00	0.01	− 0.01	− 0.08	− 0.04
Depression	0.00	− 0.05	− 0.04	0.00	− 0.01	− 0.02	− 0.05	0.00
Anger	− 0.02	− 0.09	0.09	0.03	− 0.07	0.03	− 0.07	0.01
Posttraumatic Stress	− 0.03	− 0.10	0.03	0.01	− 0.06	0.00	− 0.11	0.03
Dissociation	− 0.01	− 0.11	− 0.02	0.02	− 0.03	0.06	− 0.08	0.06
Sexual Concerns	0.01	− 0.12	− 0.10	0.04	0.03	− 0.04	0.03	0.11

*PC Restructuring* Positive Cognitive Restructuring, *D&N* Dependency and Neglect. *p <.05

The ACEs index has demonstrated positive associations with various mental health problems in adolescents involved with child welfare, including internalizing symptoms, externalizing behaviors, and trauma-related symptoms, supporting its validity for this population (Weiler et al., 2025). A cumulative risk approach was used, which aligns with the dose-response model central to ACEs research. This approach was supported by Weiler et al. (2025), who confirmed a significant linear relationship between the index and all mental health variables, with non-significant quadratic terms indicating that the cumulative model adequately captured the association between adversity and mental health in this sample. Additionally, the cumulative approach provides sufficient statistical power for moderation analyses that examining individual adversity types would not permit given our sample size.

### Analysis

All analyses were conducted using SPSS Version 29. Statistical significance was set at α = 0.05 for all analyses. Descriptive statistics and bivariate correlations were conducted to examine relationships between coping strategies, mental health problems, level of adversity, and demographics. Demographics were examined to identify potential outliers or data anomalies. Bivariate scatterplots were examined to assess the linearity of relationships between continuous variables prior to correlation analyses. Regression assumptions were assessed for all models. Multicollinearity was evaluated using Variance Inflation Factors (VIF); values ranged from 1.14 to 3.53, well below the threshold of 10 (Hair et al., 2010), with all tolerance values above 0.28, indicating no problematic multicollinearity. Independence of residuals was assessed using the Durbin-Watson statistic (1.94), within the acceptable range of 1.5–2.5. Normality of residuals was examined using Q-Q plots, and homoscedasticity was evaluated through visual inspection of scatterplots of standardized predicted values against standardized residuals. All assumptions were satisfactorily met. There were minimal missing data in the current study. Of the 245 participants, only one case (0.4%) had missing data on the coping strategy measures and one case had missing data on the Sexual Concerns subscale of the TSCC. All other TSCC subscales had complete data. Given the very low rate of missing data, listwise deletion was used in the regression analyses, which resulted in the exclusion of only one case from the analyses.

Following the preliminary analyses, hierarchical multiple regression analyses were conducted to examine whether each coping strategy was associated with each mental health outcome. Hierarchical regression was selected because it allows examination of incremental variance explained by each block of predictors while controlling for prior variables, enabling isolation of the unique contribution of interaction effects beyond main effects (Cohen et al., 2003).

The regression models were structured in three steps. In the first step, the ACEs index was included as a control variable to account for the levels of adversity experienced by the adolescents. In the second step, the five coping strategies: problem-focused coping, support-seeking, positive cognitive restructuring, distraction, and avoidance were entered as predictors to examine their direct relationship with mental health outcomes. To explore potential moderation effects, interaction terms between the ACEs index and each coping strategy were added in the third step of the models. Moderation analysis examines whether the strength or direction of the relationship between two variables differs depending on a third variable. In this study, moderation was used to test whether the associations between coping strategies and mental health outcomes varied depending on adolescents’ level of adversity exposure, specifically whether certain coping strategies may be differentially effective at different levels of cumulative ACEs. Following recommendations by Aiken and West (1991), interaction terms were entered after main effects, and all variables were centered prior to creating interaction terms to reduce multicollinearity and facilitate interpretation of coefficients.

For all significant interactions, simple slopes analysis was conducted using the regression coefficients from the hierarchical models to examine conditional effects of coping strategies at different levels of adversity (± 1 *SD* from the mean ACE index score). The ± 1 SD approach is standard practice for probing interactions in regression, as it captures meaningful variation while remaining within the observed data range for most samples (Aiken & West, 1991; Cohen et al., 2003). Simple slopes were calculated as the sum of the main effect coefficient and the interaction coefficient multiplied by the specific ACE level value. Statistical significance of simple slopes was determined using standard error calculations and t-tests.

Given the exploratory nature of the moderation analyses and the limited prior research examining adversity as a moderator of coping-outcome relationships in child welfare populations, we did not apply corrections for multiple comparisons. This approach is consistent with recommendations for exploratory research aimed at identifying patterns for future confirmatory investigation (Rothman, 1990).

## Results

### Descriptive Statistics

The study sample consisted of youth presenting with various mental health problems, with mean *t*-scores ranging from 46.02 (*SD* = 8.85) for depression to 49.41 (*SD* = 9.91) for dissociation. Other mental health problems included anxiety (*M* = 48.69, *SD* = 9.96), anger (*M* = 46.61, *SD* = 8.87), posttraumatic stress (*M* = 49.35, *SD* = 10.21), and sexual concerns (*M* = 48.95, *SD* = 14.80). The Sexual Concerns subscale, which assesses thoughts and feelings related to sexual issues including sexual preoccupation, sexual distress, and negative responses related to sexual experiences, showed considerably greater variability (*SD* = 14.80) compared to other TSCC subscales (*SD*s = 8.85–10.21), with individual scores ranging well into clinically significant levels. This variability is consistent with the heterogeneous nature of sexual experiences in child welfare-involved youth. To characterize the clinical significance of symptoms, we examined the proportion of youth scoring in the clinically significant range (*T* ≥ 65; Briere, 1996) on each TSCC subscale: Anxiety (7.8%), Depression (3.3%), Anger (4.5%), Posttraumatic Stress (11.8%), Dissociation (7.8%), and Sexual Concerns (9.4%). Posttraumatic stress and sexual concerns showed the highest rates of clinically significant symptoms, consistent with the trauma histories characteristic of child welfare-involved youth. Youth reported using various coping strategies, with distraction coping being the most frequently endorsed (*M* = 2.59, *SD* = 0.56), followed by positive cognitive restructuring (*M* = 2.55, *SD* = 0.57), problem-focused coping (*M* = 2.53, *SD* = 0.59), avoidance coping (*M* = 2.39, *SD* = 0.59), and support-seeking (*M* = 2.36, *SD* = 0.73).

### Bivariate Correlations

Table 1 presents the bivariate correlations between demographic variables, coping strategies, and mental health problems. Being African American/Black showed small positive associations with problem-focused coping and distraction coping. Other demographic variables, as well as number of referrals and previous D&N cases, did not show significant associations with coping strategies or mental health problems. Table 2 presents the bivariate correlations between ACEs and coping strategies. Overall, correlations coefficients between ACEs and coping strategies were generally small and predominantly non-significant. Notable exceptions included sexual abuse, which showed significant negative associations with problem-focused coping and positive cognitive restructuring, and peer emotional bullying, which was also negatively related to positive cognitive restructuring. Finally, the ACEs Index was significantly negatively correlated with positive cognitive restructuring. No other significant correlations emerged between the ACEs Index and coping strategies.

**Table 2:** Correlations Between Adverse Childhood Experiences and Coping Strategies

Variable	Problem-focused	Support-Seeking	PC Restructuring	Distraction Coping	Avoidance Coping
Physical Abuse	0.03	− 0.01	− 0.03	− 0.04	− 0.0
Sexual Abuse	− 0.14*	− 0.09	− 0.16*	− 0.03	0.01
Physical Neglect	0.03	− 0.02	− 0.01	0.03	− 0.06
Termination of Parental Rights	0.04	0.04	− 0.04	0.03	− 0.06
Witnessing Domestic Violence	− 0.08	− 0.04	− 0.11	− 0.11	− 0.04
Caregiver Transitions	− 0.12	− 0.04	− 0.10	− 0.06	− 0.09
School Changes	− 0.05	0.00	− 0.04	− 0.02	− 0.03
Community Violence Exposure	0.01	0.05	0.02	0.12	0.08
Discrimination-Based Violence	0.03	0.03	0.03	0.04	− 0.01
Peer Emotional Bullying	− 0.07	0.05	− 0.15*	− 0.01	− 0.03
Peer Physical Bullying	0.03	0.08	0.04	0.07	0.06
Emotional/Relational Dating Violence	− 0.08	− 0.09	− 0.11	0.00	0.04
Sexual/Physical Dating Violence	− 0.09	0.00	− 0.07	− 0.04	0.03
Cyberbullying	− 0.12	− 0.02	− 0.06	0.00	0.07
ACEs Index (14-item)	− 0.11	− 0.02	− 0.14*	− 0.01	− 0.02

*N* = 242–244. *PC Restructuring* Positive Cognitive Restructuring. *p <.05

### Hierarchical Regression Analysis

As shown in Table 3, after accounting for the effect of ACEs, the coping strategies showed distinct patterns of association with specific mental health problems. Problem-focused coping was negatively associated with anger symptoms. Support-seeking showed no significant associations with any mental health problems. Positive cognitive restructuring was negatively related to anxiety, depression, and posttraumatic stress. Distraction coping was positively associated with depression and anger. Avoidance coping was positively related to anxiety, depression, posttraumatic stress, and dissociation. The addition of the interaction terms in Step 3 explained additional variance in each model: Anxiety (Δ*R*² = 0.024), Depression (Δ*R*² = 0.035), Anger (Δ*R*² = 0.027), Posttraumatic Stress (Δ*R*² = 0.026), Dissociation (Δ*R*² = 0.035), and Sexual Concerns (Δ*R*² = 0.054, *p* =.007). The Sexual Concerns model was the only model in which the interaction block reached statistical significance. Full model *R*² values ranged from 0.202 (Anxiety) to 0.294 (Anger).

**Table 3:** Hierarchical Regression Analyses of Youth Mental Health Problems

Variables	Anxiety		Depression		Anger		PTS		Dissociation		SC	
	β	*R*²/*p*	β	*R*²/*p*	β	*R*²/*p*	β	*R*²/*p*	β	*R*²/*p*	β	*R*²/*p*
Step 1:		0.15***		0.16***		0.19***		0.20***		0.15***		0.17***
ACEs	0.39***	< 0.001	0.40***	< 0.001	0.45***	< 0.001	0.45***	< 0.001	0.40***	< 0.001	0.42***	< 0.001
Step 2:		0.16***		0.20***		0.25***		0.22***		0.19***		0.17***
ACEs	0.36***	< 0.001	0.36***	< 0.001	0.40***	< 0.001	0.42***	< 0.001	0.38***	< 0.001	0.43***	< 0.001
Problem-focused Coping	0.04	0.684	− 0.09	0.345	− 0.22*	0.015	0.01	0.899	− 0.06	0.506	− 0.05	0.596
Support-seeking	0.05	0.557	0.02	0.775	0.04	0.612	0.02	0.846	− 0.10	0.213	− 0.02	0.817
PC Restructuring	− 0.24*	0.016	− 0.24*	0.012	− 0.17	0.061	− 0.26*	0.007	− 0.07	0.456	0.09	0.354
Distraction Coping	− 0.01	0.920	0.15*	0.040	0.21**	0.004	0.05	0.453	0.11	0.126	− 0.03	0.717
Avoidance Coping	0.17*	0.022	0.18*	0.012	0.12	0.093	0.21**	0.003	0.21**	0.005	0.09	0.252
Step 3:		0.16***		0.21***		0.26***		0.23***		0.21***		0.21***
ACEs	0.33***	< 0.001	0.33***	< 0.001	0.36***	< 0.001	0.38***	< 0.001	0.36***	< 0.001	0.42***	< 0.001
Problem-focused Coping	0.03	0.736	− 0.11	0.222	− 0.22*	0.018	− 0.01	0.947	− 0.09	0.365	− 0.06	0.503
Support-seeking	0.06	0.460	0.04	0.610	0.03	0.706	0.02	0.768	− 0.10	0.213	− 0.04	0.650
PC Restructuring	− 0.25*	0.013	− 0.23*	0.016	− 0.18	0.060	− 0.25*	0.010	− 0.06	0.557	0.10	0.292
Distraction Coping	− 0.03	0.746	0.13	0.094	0.22**	0.003	0.04	0.553	0.11	0.136	− 0.01	0.949
Avoidance Coping	0.21**	0.007	0.22**	0.004	0.13	0.071	0.23**	0.002	0.23**	0.002	0.11	0.144
ACEs × Problem-focused	− 0.02	0.840	− 0.11	0.280	− 0.21*	0.033	− 0.16	0.118	− 0.22*	0.035	− 0.25*	0.016
ACEs × Support-seeking	0.07	0.350	0.04	0.573	0.19*	0.011	0.10	0.174	0.02	0.786	− 0.04	0.618
ACEs × PC Restructuring	− 0.02	0.857	− 0.02	0.867	0.02	0.836	0.03	0.778	0.16	0.137	0.33**	0.003
ACEs × Distraction	− 0.03	0.677	0.06	0.420	0.05	0.485	0.07	0.352	0.01	0.865	− 0.10	0.192
ACEs × Avoidance	0.16*	0.046	0.20*	0.011	0.06	0.416	0.13	0.107	0.14	0.082	0.08	0.295

*PTS* Posttraumatic Stress, *SC* Sexual Concerns, *PC* Restructuring = Positive Cognitive Restructuring. Standardized betas (*β*) and adjusted *R*² are reported. The R²/p column displays adjusted R² with significance of F-change for step rows and exact p values for predictor rows. *p <.05. **p <.01. ***p <.001

**Fig. 1:**
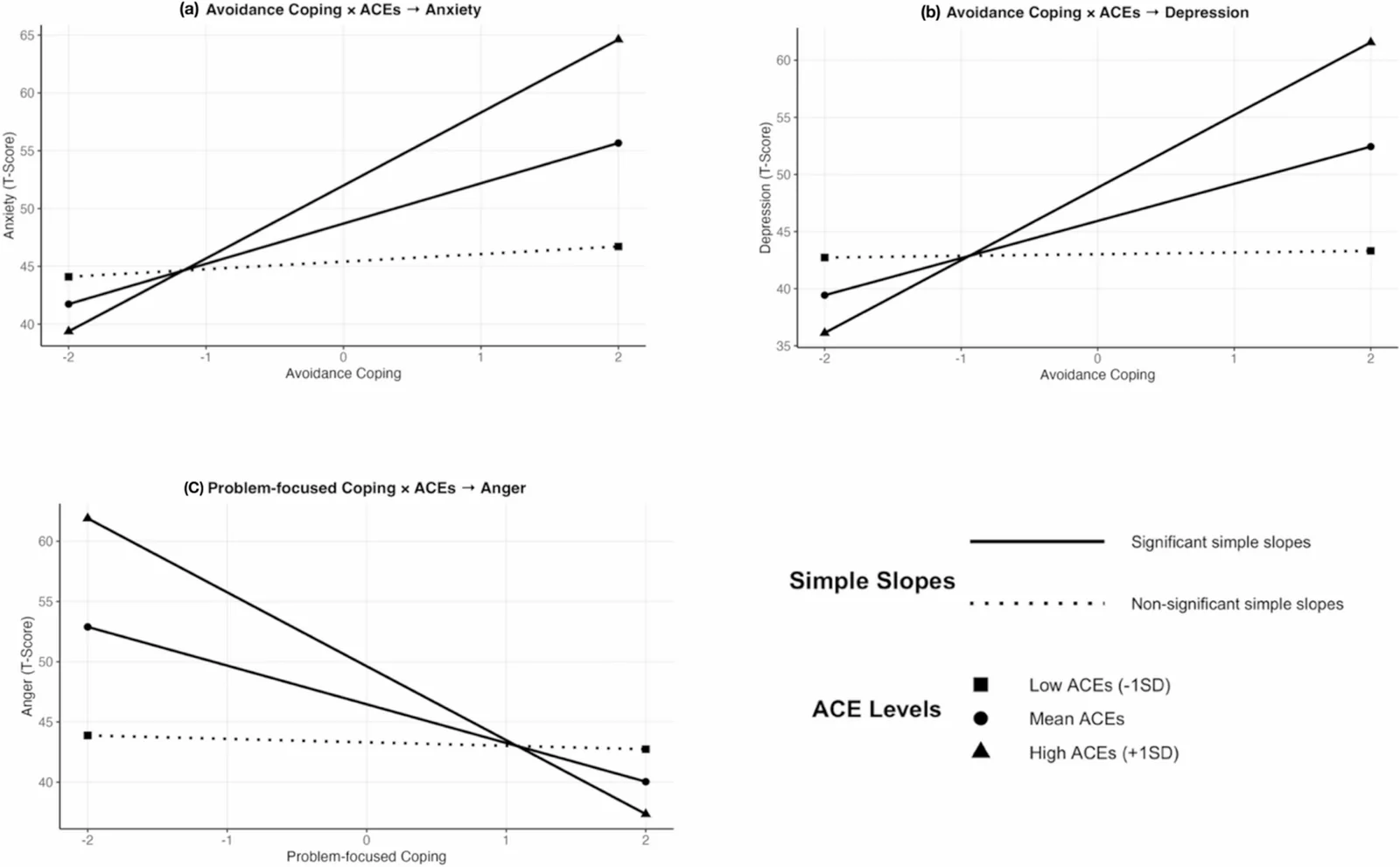

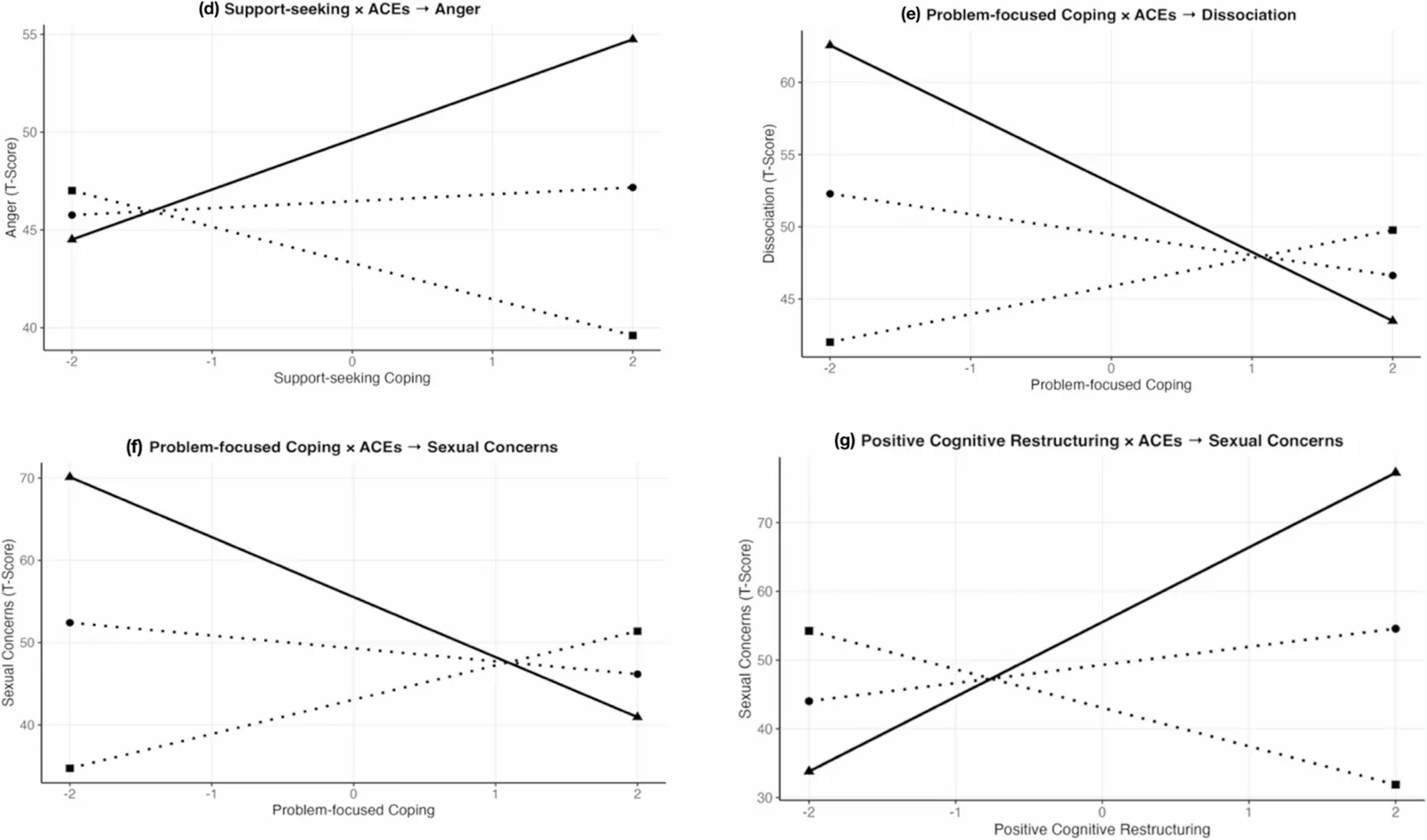
Moderation Effects of Childhood Adversity on Coping-Mental Health Relationships. (**a**) Avoidance was significantly positively associated with anxiety at high (*b* = 6.31, *p*=.001) and average (*b* = 3.48, *p* =.007) ACE levels, but not at low ACE levels (*b* = 0.66, *p* =.732). (**b**) Avoidance was significantly positively associated with depression at high (*b* = 6.36, *p* <.001) and average (*b* = 3.25, *p* =.004) ACE levels, but not at low ACE levels (*b* = 0.15, *p* =.930). Problem-focused coping was significantly negatively associated with anger at high (*b* = −6.14,*p* =.002) and average (*b* = −3.21, *p* =.018) ACE levels, but not at low ACE levels (*b* = −0.29, *p* =.882). (**c**) Problem-focused coping was significantly negatively associated with anger at high (*b* = −6.14,*p* =.002) and average (*b* = −3.21, *p* =.018) ACE levels, but not at low ACE levels (*b* = −0.29, *p* =.882). (**d**) Support-seeking was significantly positively associated with anger at high ACE levels only (*b* = 2.56, *p* =.045), with no significant associations at average (*b* = 0.35, *p* =.706) or low (*b* = −1.85, *p* =.146) ACE levels. (**e**) Problem-focused coping was significantly negatively associated with dissociation at high ACE levels only (*b* = −4.77, *p* =.033), with no significant associations at average (*b* = −1.42, *p* =.364) or low (*b* = 1.94,*p* =.383) ACE levels. Figures display predicted TSCC T-scores (y-axis) by coping strategy use (x-axis, rated 1–4) at three levels of adverse childhood experiences (ACEs): low (1 *SD* below the mean ≈ 1 ACE), average (at the mean ≈ 3 ACEs), and high (1 *SD* above the mean ≈ 5 ACEs). Higher T-scores indicate greater symptom severity. Asterisks indicate slopes significantly different from zero (*p* <.05). *N* = 244 (243 for Sexual Concerns). (**f**) Problem-focused coping was significantly negatively associated with sexual concerns at high ACE levels only (*b* = −7.29, *p* =.029), with no significant associations at average (*b* = −1.56, *p* =.503) or low (*b* = 4.17,*p* =.211) ACE levels. (**g**) Positive cognitive restructuring was significantly positively associated with sexual concerns at high ACE levels only (*b* = 10.86, *p* =.003), with no significant associations at average (*b* = 2.64, *p* =.292) or low (*b*= −5.59, *p* =.130) ACE levels

Additionally, several significant moderation effects were observed across the various models (see Fig. 1). The relationship between problem-focused coping and mental health problems was moderated by ACEs for anger (*B* = −18.83, *SE* = 8.80, *β* = − 0.21, *p* =.033), dissociation (*B* = −21.60, *SE* = 10.18, *β* = − 0.22, *p* =.035), and sexual concerns (*B* = −36.89, *SE* = 15.22, *β* = − 0.25, *p* =.016). Simple slopes analysis revealed that problem-focused coping showed increasingly strong negative associations with anger as ACE levels increased: significant at high (*b* = −6.14, *p* =.002, 95% CI [−9.91, −2.36]) and average ACE levels (*b* = −3.21, *p* =.018, 95% CI [−5.86, −0.56]), but not at low ACE levels (*b* = −0.29, *p* =.882, 95% CI [−4.07, 3.49]). For dissociation, problem-focused coping showed significant negative associations only at high ACE levels (*b* = −4.77, *p* =.033, 95% CI [−9.14, −0.40]), with no significant associations at average (*b* = −1.42, *p* =.364, 95% CI [−4.49, 1.65]) or low ACE levels (*b* = 1.94, *p* =.383, 95% CI [−2.44, 6.31]). Regarding sexual concerns, problem-focused coping was significantly negatively associated with symptoms at high ACE levels (*b* = −7.29, *p* =.029, 95% CI [−13.83, −0.75]) but showed no significant associations at average (*b* = −1.56, *p* =.503, 95% CI [−6.16, 3.03]) or low ACE levels (*b* = 4.17, *p* =.211, 95% CI [−2.38, 10.70]).

The support-seeking × ACEs interaction was significant for anger (*B* = 14.19, *SE* = 5.52, *β* = 0.19, *p* =.011). Support-seeking showed significant positive associations with anger symptoms only at high ACE levels (*b* = 2.56, *p* =.045, 95% CI [0.06, 5.06]), with no significant associations at average (*b* = 0.35, *p* =.706, 95% CI [−1.49, 2.20]) or low ACE levels (*b* = −1.85, *p* =.146, 95% CI [−4.35, 0.65]). The positive cognitive restructuring × ACEs interaction was significant for sexual concerns (*B* = 52.98, *SE* = 17.46, *β* = 0.33, *p* =.003), the strongest individual interaction effect observed. Positive cognitive restructuring demonstrated significant positive associations with sexual concerns only at high ACE levels (*b* = 10.86, *p* =.003, 95% CI [3.60, 18.13]), with no significant associations at average (*b* = 2.64, *p* =.292, 95% CI [−2.28, 7.55]) or low ACE levels (*b* = −5.59, *p* =.130, 95% CI [−12.85, 1.67]). Distraction coping showed no significant moderation effects with ACEs across any mental health problems.

The avoidance × ACEs interaction was significant for anxiety (*B* = 18.20, *SE* = 9.09, *β* = 0.16, *p* =.046) and depression (*B* = 20.01, *SE* = 7.84, *β* = 0.20, *p* =.011). For anxiety, avoidance showed significant positive associations at high (*b* = 6.31, *p* =.001, 95% CI [2.55, 10.07]) and average ACE levels (*b* = 3.48, *p* =.007, 95% CI [0.94, 6.02]), but not at low ACE levels (*b* = 0.66, *p* =.732, 95% CI [−3.11, 4.42]). Similarly, avoidance was significantly positively associated with depression at high (*b* = 6.36, *p* <.001, 95% CI [3.11, 9.61]) and average ACE levels (*b* = 3.25, *p* =.004, 95% CI [1.06, 5.44]), but not at low ACE levels (*b* = 0.15, *p* =.930, 95% CI [−3.10, 3.39]).

## Discussion

This study examined relationships between coping strategies (i.e., problem-focused coping, support-seeking coping, positive cognitive restructuring, distraction coping, and avoidance coping) and mental health problems (i.e., anxiety, depression, anger, posttraumatic stress, dissociation, and sexual concerns) among youth involved in the child welfare system. This study focused on how these relationships vary by level of adversity exposure, recognizing that associations between coping and mental health may depend on the accumulation of adverse experiences. The findings provide several insights regarding the complex relationships between adversity, coping, and mental health in this population. Within the Responses to Stress framework (Connor-Smith et al., 2000), PCEC and SCEC strategies are theorized to be adaptive because they involve active efforts to address the source of stress or to adapt to its presence, while DC strategies are considered maladaptive because they involve withdrawal from the stressor without resolution. However, this framework was developed primarily with general adolescent populations, leaving open the question of whether these theoretical predictions hold for youth whose stress exposure is chronic, cumulative, and often uncontrollable. The current findings both support and complicate the RTS framework’s predictions in important ways.

### Hypothesis 1: Primary Control Engagement Coping and Mental Health

The hypothesis that PCEC strategies would be negatively associated with mental health problems received partial support. Problem-focused coping showed a significant negative association with anger symptoms, suggesting that active problem-solving approaches are associated with lower anger levels. This finding aligns with previous research demonstrating benefits of problem-solving approaches in foster care settings, where youth who approach problems directly show lower internalizing and externalizing symptoms (Huffhines et al., 2020). However, this protective association was not present across all mental health problems, indicating that problem-focused coping may be relevant for externalizing symptoms like anger, rather than serving as a universal protective factor.

Support-seeking, the second PCEC strategy examined, did not show the expected negative associations with mental health problems. Instead of direct associations, support-seeking only demonstrated relationships with mental health problems when examined in interaction with ACEs (discussed further under Hypothesis 4). The absence of main effects for support-seeking contrasts with general adolescent coping literature but aligns with research specific to child welfare populations. Previous studies have shown that support-seeking effectiveness is heavily moderated by available resources, with youth who have limited social support experiencing increased distress when attempting to seek help that is unavailable or unreliable (Grey et al., 2015). Additionally, child welfare involvement itself may compromise youth’s orientation toward help-seeking, as placement instability has been identified as the strongest predictor of reduced social network orientation (Seita et al., 2016). The potentially disrupted and unstable social networks characteristic of child welfare involvement may render support-seeking strategies less directly effective than in community samples. This contrasts with studies of general adolescent populations where social networks are typically more stable and responsive (Compas et al., 2001), highlighting that the effectiveness of support-seeking depends not only on the strategy itself but on the social context in which it is deployed.

Together, these PCEC findings suggest that the RTS framework’s prediction of protective associations for engagement coping strategies is partially supported but contingent on the specific strategy and the availability of external resources. Problem-focused coping aligns with framework predictions for anger, but the null findings for support-seeking suggest that the framework’s assumptions about engagement coping may not fully account for the constrained social environments of child welfare-involved youth.

### Hypothesis 2: Secondary Control Engagement Coping and Mental Health

The hypothesis that SCEC strategies would be negatively associated with mental health problems received substantial support. Positive cognitive restructuring demonstrated significant negative associations with anxiety, depression, and posttraumatic stress symptoms. These findings were somewhat unexpected given previous research suggesting that maltreatment exposure affects fundamental cognitive processes including attention, information integration, and decision-making (Dvir et al., 2014; Teicher et al., 2016). Additionally, research with emancipated foster youth has shown that cognitive reframing can be problematic when employed by youth with limited cognitive resources (Grey et al., 2015). However, the current results suggest that when youth are able to employ positive cognitive restructuring, it demonstrates protective associations across multiple internalizing symptom domains. The associations were specific to internalizing symptoms (anxiety, depression, posttraumatic stress) but were not observed for anger, dissociation, or sexual concerns. This specificity may reflect differential mechanisms underlying various symptom types. Internalizing symptoms often involve cognitive components such as negative self-evaluation, hopelessness, and catastrophic thinking (Clarke, 2006), which may be more directly addressable through cognitive restructuring approaches. In contrast, dissociative symptoms involve alterations in consciousness and disconnection from thoughts and feelings potentially making them less responsive to conscious cognitive strategies (Cavicchioli et al., 2021). The discrepancy between our findings and prior research suggesting cognitive reframing is problematic for maltreated youth (Grey et al., 2015) may reflect important differences in sample and measurement. Grey et al. (2015) studied emancipated foster youth, older and no longer receiving services, whereas the current sample comprises younger adolescents, many of whom are actively receiving child welfare services. Additionally, our measure assessed general coping tendencies rather than coping with specific traumatic events, which may capture different processes than studies examining trauma-specific coping. These findings are largely consistent with the RTS framework’s prediction that SCEC strategies are protective for mental health. However, the specificity of these associations to internalizing symptoms, and the reversal observed for sexual concerns at high adversity levels (discussed under Hypothesis 4), suggests that the framework’s predictions may need refinement to account for the type of mental health outcome and level of adversity exposure.

### Hypothesis 3: Disengagement Coping and Mental Health

The hypothesis that DC strategies would be positively associated with mental health problems was largely supported. However, these findings present a more straightforward pattern than the complex picture suggested by previous research. Avoidance coping showed consistent positive associations with anxiety, depression, posttraumatic stress, and dissociation. It is important to note that due to the cross-sectional nature of this study we cannot conclude that avoidance coping leads to more mental health problems. A plausible alternative hypothesis is that youth experiencing higher levels of anxiety, depression, posttraumatic stress, and dissociation may be more likely to engage in avoidance coping as these symptoms themselves often involve avoidant tendencies (e.g., avoidance of trauma reminders in PTSD, social withdrawal in depression). While previous research has identified potential protective functions of disengagement strategies, particularly for youth with extensive maltreatment histories facing uncontrollable stressors (Huffhines et al., 2020), the current findings suggest null or maladaptive associations in this sample. Distraction coping showed positive associations with depression and anger but not with anxiety, posttraumatic stress, dissociation, or sexual concerns. These associations may reflect several possible mechanisms, though causal interpretations are limited by the study design. From a theoretical perspective, while DC strategies may provide temporary relief from distressing emotions or situations, their habitual use is associated with the accumulation of unresolved stressors and the prevention of emotional processing necessary for adaptation (Aldao et al., 2010; Pacella et al., 2011). However, it is also plausible that youth experiencing significant psychological distress naturally gravitate toward avoidant strategies to manage overwhelming symptoms. The bidirectional nature of these relationships highlights the complexity of coping processes and underscores the need for longitudinal research to clarify developmental patterns and intervention research to identify causal mechanisms. These findings are consistent with the RTS framework’s conceptualization of disengagement coping as maladaptive. However, the moderation findings indicate that this association is not uniform—it strengthens at higher adversity levels—suggesting that the framework would benefit from incorporating adversity level as a moderating factor rather than treating disengagement as universally maladaptive.

### Hypothesis 4: Adversity as a Moderator of Coping-Mental Health Relationships

The hypothesis that ACEs would moderate relationships between coping strategies and mental health problems was supported through several significant interaction effects. While these analyses may be underpowered to detect small effects, the observed patterns suggest nuances in how coping strategies relate to mental health outcomes across different levels of adversity exposure. The temporal dimension of ACEs is critical for understanding these moderation effects. Some participants’ ACEs occurred years prior to data collection at ages 12–15, with some experiencing adversity in early childhood (Weiler et al., 2025), such that youth may be coping with the residual effects of past traumas and ongoing life challenges. Strategies that may have been protective during active trauma exposure may function differently when employed years later. These findings align with theoretical frameworks suggesting that as adversity accumulates, the relationship between coping strategies and mental health outcomes may shift, with certain strategies becoming less effective or counterproductive in the context of severe or chronic adversity (Huffhines et al., 2020; Perzow et al., 2021). This shifting effectiveness may reflect neurobiological impacts of repeated stress exposure that dysregulate stress response systems and affect emotional processing and regulation capacities (Dvir et al., 2014; McLaughlin & Lambert, 2017), potentially altering whether and how youth cope.

For problem-focused coping, significant interactions emerged for anger, dissociation, and sexual concerns. At high ACE levels, problem-focused coping showed strong negative associations with these variables, while at low ACE levels, no significant associations were observed. For youth with lower ACE levels, the lack of association may reflect that these youth face fewer stressors requiring intensive coping effort. Their mental health symptoms may be determined more by other factors than by their use of problem-focused coping strategies. Alternatively, the lower overall symptom levels in this group may create a floor effect, limiting variability needed to detect associations.

Support-seeking showed a significant interaction with ACEs for anger symptoms, with contrasting patterns at different adversity levels. At low ACE levels, there was a trend for support-seeking to be associated with reduced anger, while at high ACE levels, it was associated with increased anger symptoms. This finding aligns with Grey et al.’s (2015) work suggesting that social support quality and availability may vary significantly for youth with different adversity histories. Youth with extensive trauma histories may seek support from social networks that are themselves overwhelmed, traumatized, or unable to provide effective guidance, leading to frustration and increased anger when their support-seeking efforts are unsuccessful or result in poor advice. When youth with high adversity exposure attempt to seek support but encounter rejection, invalidation, or inadequate responses, this may trigger anger responses related to past experiences of unmet needs or broken trust. Furthermore, youth with extensive trauma histories may be more likely to seek support for complex, severe problems that overwhelm potential supporters, leading to cycles of disappointment and anger.

Positive cognitive restructuring demonstrated an unexpected interaction pattern with sexual concerns. Rather than showing protective associations, positive cognitive restructuring was associated with increased sexual concerns among youth with high ACEs level. This counterintuitive finding may reflect the particular challenges of processing sexual trauma. Because the moderation analysis used the cumulative ACEs index rather than individual adversity types, it cannot be determined whether this association is driven specifically by sexual abuse experiences or by cumulative adversity burden more broadly. With this caveat in mind, several possible explanations warrant consideration. First, sexual abuse fundamentally violates bodily autonomy and personal boundaries in ways that may be impossible to reframe positively without minimizing the severity of the experience (Cole & Putnam, 1992). Youth with sexual abuse histories may find that attempts to reframe sexual trauma triggers increased distress, as these experiences may be particularly difficult to view in positive terms. Second, research by Shipman et al. (2000) found that girls who experienced sexual abuse demonstrated lower emotional understanding and expected more interpersonal conflict when expressing negative emotions compared to non-maltreated peers. While this study did not examine cognitive restructuring directly, the findings suggest that sexual abuse disrupts fundamental emotional processing abilities. These disruptions may make cognitive restructuring particularly challenging or distressing when applied to sexual trauma content. Youth attempting to cognitively reframe their experiences may encounter these underlying emotional processing deficits, leading to increased distress rather than symptom relief. It is also important to note that this study measured positive cognitive restructuring specifically, which emphasizes finding positive aspects or “silver linings” in difficult situations (e.g., “You reminded yourself that things could be worse”). For youth with extensive adversity histories, particularly those who have experienced sexual abuse, attempting to find positive aspects of genuinely harmful experiences may feel invalidating or may require minimizing the severity of what occurred. Broader cognitive restructuring approaches that emphasize developing realistic, balanced interpretations—rather than purely positive ones—may function differently. This interpretation is supported by the finding that the positive cognitive restructuring × ACEs interaction was significant only for Sexual Concerns (*B* = 52.98, *p* =.003) and not for other mental health outcomes, where positive cognitive restructuring showed consistently protective main effects. Future research should examine whether cognitive restructuring approaches that emphasize realistic reappraisal, rather than positive reframing, show different patterns of association with mental health outcomes in high-adversity populations.

Avoidance coping showed significant positive interactions with anxiety and depression at average and high ACE levels, but not low ACE levels. These findings somewhat contrast with previous research suggesting that avoidance strategies might serve protective functions for youth with extensive adversity exposure, particularly when facing severe, uncontrollable stressors (Huffhines et al., 2020). The current results suggest that avoidance becomes increasingly maladaptive as adversity levels rise. Several specific factors may account for the discrepancy with prior findings. First, Huffhines et al. (2020) examined youth who had already been removed from their homes, whereas the current sample includes a substantial proportion (55.1%) still living at home. Youth remaining in their homes may face ongoing adversity exposure, making avoidance less effective as a coping strategy because the stressor is not time-limited or past. Second, the types of adversities captured by our expanded 14-item ACEs index, which includes peer violence, school instability, and caregiver transitions alongside traditional maltreatment types, may differ from the adversities measured in prior studies, and avoidance may function differently depending on whether stressors are acute versus chronic or controllable versus uncontrollable. Third, the current study examined specific symptom domains (e.g., anxiety, depression) rather than broad internalizing composites, which may reveal patterns obscured in prior research using aggregated outcome measures. Notably, distraction coping showed direct associations with depression and anger but did not demonstrate any significant moderation effects with ACEs, suggesting that its associations with mental health problems are not significantly different across adversity levels.

Taken together, these moderation effects point to possible theoretical implications for the RTS framework that warrant further investigation. While the framework categorizes strategies as broadly adaptive (PCEC, SCEC) or maladaptive (DC), the current findings suggest that these categorizations may not be absolute. The effectiveness of coping strategies, and in some cases, the direction of their associations with mental health, may depend on the level of cumulative adversity youth have experienced. If replicated, these findings suggest that coping frameworks should move beyond static categorizations toward more dynamic models that account for contextual moderators, particularly adversity exposure.

### Strengths and Limitations

This study has several notable strengths. The relatively large and diverse sample of adolescents involved in the child welfare system enhances the generalizability of findings to this population. The use of an expanded ACEs Index specifically developed for child welfare-involved youth provides a more comprehensive assessment of adversity than traditional measures, capturing experiences particularly relevant to this population. Furthermore, the examination of moderation effects represents an advancement in understanding how coping associations may vary according to adversity level, moving beyond simplistic models that assume universal patterns of coping-mental health relationships.

Several limitations, however, should be considered when interpreting the findings. The cross-sectional design precludes causal inference. We cannot establish temporal precedence, it is equally plausible that youth with elevated mental health symptoms may engage in different coping patterns as a response to their distress, rather than coping strategies influencing symptom levels. For example, the positive association between avoidance coping and internalizing symptoms could reflect youth with heightened anxiety and depression gravitating toward avoidant strategies, rather than avoidance driving symptom increases. Similarly, the unexpected association between positive cognitive restructuring and sexual concerns at high ACE levels could reflect youth with elevated sexual concerns engaging in more cognitive reframing efforts in response to these concerns. Bidirectional relationships and unmeasured third variables may also account for observed associations. Although the study is grounded in theoretical frameworks (Connor-Smith et al., 2000; Compas et al., 2001) and longitudinal research in general adolescent populations supporting coping as an antecedent of mental health outcomes (Compas et al., 2017), future longitudinal research is needed to confirm these directional relationships specifically within child welfare populations.

While the cumulative ACEs approach effectively captures the overall burden of adversity, it does not distinguish between types, frequency, or severity of individual adverse experiences. As a result, we cannot determine whether specific adversity types, such as sexual abuse, may differentially moderate coping-outcome relationships. Future research with larger samples could examine whether particular types of adversity play distinct roles in shaping coping effectiveness. It is also important to note that all participants in the current study had open child welfare cases, meaning they had experienced at least some level of adversity. A score of 0 on the ACEs index does not indicate an absence of adversity but rather that a youth did not endorse any of the 14 specific indicators that were empirically associated with mental health outcomes in this population (Weiler et al., 2025). Of the 21 candidate ACEs initially considered, seven were not retained in the final index because they were not significantly positively correlated with mental health variables in this sample (for full details, see Weiler et al., 2025).

The number of moderation tests conducted across six mental health outcomes and five coping strategies increases the possibility of Type I error. Given the exploratory nature of this research and the limited prior work examining adversity as a moderator of coping-outcome relationships in child welfare populations, we did not apply formal corrections for multiple comparisons, prioritizing the identification of potentially meaningful patterns for future confirmatory investigation (Rothman, 1990). Findings from individual interaction terms should therefore be interpreted with appropriate caution.

Additionally, the sample size was determined based on the primary study’s main objectives rather than an a priori power calculation specifically for moderation analyses (Aguinis et al., 2005). Consequently, these interaction models may be underpowered to detect small effects. These findings should therefore be treated as exploratory, providing a preliminary signal that warrants replication in fully powered studies.

The reliance on self-report measures for both coping strategies and mental health symptoms may introduce shared method variance. However, self-report is considered essential for assessing internal processes such as coping strategies, which involve cognitive and volitional efforts not directly observable by others (Compas et al., 2001). Additionally, youth self-report is particularly valuable for internalizing symptoms, as these internal experiences are less accessible to external observers, and self-ratings may require heavier weighting for constructs such as depressive symptoms compared to more observable behaviors (De Los Reyes et al., 2015; Martel et al., 2017). Multi-informant approaches incorporating caregiver or teacher reports could strengthen future research by reducing shared method variance and providing complementary perspectives on youth functioning.

Regarding generalizability, because participants were required to live within a 35-minute drive of the program site, youth in more rural or geographically isolated areas are not represented. These youth may face different barriers to services and social support, and their coping patterns and mental health profiles could differ from the current sample, limiting generalizability to the broader child welfare population.

Finally, bivariate correlations between individual ACEs and coping strategies were generally small and predominantly non-significant. This pattern is not unexpected for several reasons. First, the cumulative ACEs index, rather than individual adversity items, served as the moderator in this study, and the index was significantly correlated with positive cognitive restructuring. The cumulative approach captures the combined burden of adversity, which may be more consequential for coping processes than any single experience. Second, the dichotomous nature of individual ACE items (experienced vs. not experienced) limits variability and reduces the magnitude of possible correlations. Third, the coping measure assesses general coping tendencies rather than responses to specific adverse events, which may attenuate associations with individual adversity types. Future research using more detailed adversity assessments that capture frequency, severity, and developmental timing could yield stronger associations with coping patterns.

### Implications and Future Direction

Although these findings are exploratory and require replication, they point to several preliminary considerations for clinical intervention with adolescents involved in the child welfare system. One consideration is that practitioners should assess adversity history when implementing coping-based interventions, as the current findings suggest that strategies effective for general adolescent populations may not function equivalently for youth with extensive adversity histories. The consistent negative associations between SCEC strategies (particularly positive cognitive restructuring) and multiple mental health outcomes (anxiety, depression, and posttraumatic stress) suggest that interventions targeting cognitive coping skills may be particularly beneficial for these specific symptom domains. Trauma-focused cognitive-behavioral therapy and other approaches that teach youth to recognize and reframe negative thought patterns could be especially effective for reducing internalizing symptoms (Compas et al., 2017; McLaughlin et al., 2014). However, the interaction between positive cognitive restructuring and adversity for sexual concerns warrants careful consideration. The finding that positive cognitive restructuring was associated with increased sexual concerns at high adversity levels suggests that for youth with extensive adversity exposure, particularly those with sexual trauma, cognitive reframing approaches may need careful implementation or may be contraindicated. This aligns with trauma literature suggesting that some symptoms, particularly those related to sexual trauma, may require specialized trauma-focused intervention rather than general cognitive coping strategies (Caouette et al., 2023; Shipman et al., 2000). Clinicians working with high-ACE youth may benefit from emphasizing realistic cognitive reappraisal, helping youth develop accurate, balanced interpretations of their experiences, rather than purely positive reframing, which may feel invalidating or impossible for youth processing genuinely harmful experiences. More broadly, practitioners should monitor for unintended effects when teaching traditionally “adaptive” coping strategies to youth with complex trauma histories, as the current findings suggest these strategies do not function uniformly across adversity levels.

Avoidance coping was associated with increased mental health problems. This association was strongest for youth with high adversity exposure. Avoidant coping showed consistent positive associations with anxiety, depression, posttraumatic stress, and dissociation. While avoidance may serve an adaptive function in the immediate aftermath of trauma, the current findings suggest that reliance on avoidance may be associated with higher symptom levels. Intervention approaches should include components that address avoidance tendencies while simultaneously building alternative coping skills. This might involve trauma-informed approaches that acknowledge avoidance as an initially adaptive response to overwhelming experiences while gradually introducing more engagement-oriented strategies and addressing underlying emotional expression deficits that can compromise interpersonal coping efforts (Ford et al., 2012; Pacella et al., 2011). The stronger relationship between avoidance and mental health problems at higher levels of adversity suggests that interventions may need to be tailored according to adversity exposure, with more intensive support provided for youth with extensive trauma histories (Aldao et al., 2010; Compas et al., 2017). The finding that support-seeking was associated with increased anger at high ACE levels also has clinical implications. Practitioners implementing interpersonal or support-based interventions with high-adversity youth should consider whether adequate, reliable support networks are in place before encouraging active help-seeking, and may need to simultaneously strengthen youths’ social resources and help them develop realistic expectations for support interactions. The association between avoidance coping and diverse symptoms including PTSD (PTS and dissociation) raises important considerations about the overlap between coping behaviors and symptom manifestations. Avoidance represents both a voluntary coping strategy and a core symptom of PTSD, suggesting the need for careful assessment to distinguish between coping efforts and symptoms expression in clinical contexts.

These exploratory findings underscore the potential importance of trauma-informed services within child welfare systems that address both the development of adaptive coping skills that lead to positive youth development and wellbeing. Additionally, the results highlight the importance of comprehensive assessment approaches that consider both the level of adversity experienced and the coping strategies employed when developing intervention plans for youth in the child welfare system (Kim & Cicchetti, 2010; Taussig et al., 2019). Future research should extend these findings through longitudinal designs to clarify temporal relationships and intervention studies to establish causal mechanisms between coping strategies and mental health problems. Additionally, studies that incorporate multiple assessment methods and examine coping within specific situational contexts relevant to child welfare-involved youth would provide more nuanced understanding (Luecken et al., 2009; Scott Heller et al., 1999). Research examining coping within specific situational contexts relevant to child welfare-involved youth—such as placement transitions, court proceedings, or family visits—would provide more nuanced understanding of how coping functions in this population’s daily experiences. This is particularly important given that placement instability has been identified as uniquely problematic for child welfare-involved youth, systematically undermining trust and social network orientation that are foundational for many coping strategies (Seita et al., 2016; Unrau et al., 2008). Additionally, investigating the mechanisms through which adversity influences coping-mental health associations could inform more targeted intervention development.

## Conclusion

This study adds to the literature by examining how coping strategies relate to mental health outcomes among adolescents with child welfare involvement and whether these associations vary by cumulative adversity exposure. The findings reveal that positive cognitive restructuring was associated with lower anxiety, depression, and posttraumatic stress symptoms, while avoidance and distraction coping were associated with higher symptoms across multiple domains. Notably, adversity levels moderated several of these relationships, with problem-focused coping showing stronger protective associations at high adversity levels for certain outcomes, while support-seeking and positive cognitive restructuring demonstrated unexpected associations with increased symptoms among youth with extensive trauma histories. These context-dependent patterns suggest that coping effectiveness cannot be assumed to be universal in this population. As interventions continue to be developed for child welfare-involved youth, these findings highlight the need for trauma-informed approaches that consider both cumulative adversity exposure and the complex ways coping strategies may function differently based on youths’ trauma histories and available resources.

## Data Availability

No datasets were generated or analysed during the current study.
